# Interleukin-17A Contributes to the Control of *Streptococcus pyogenes* Colonization and Inflammation of the Female Genital Tract

**DOI:** 10.1038/srep26836

**Published:** 2016-05-31

**Authors:** Alison J. Carey, Jason B. Weinberg, Suzanne R. Dawid, Carola Venturini, Alfred K. Lam, Victor Nizet, Michael G. Caparon, Mark J. Walker, Michael E. Watson, Glen C. Ulett

**Affiliations:** 1 School of Medical Sciences, and Menzies Health Institute Queensland, Griffith University, Parklands, QLD 4222, Australia; 2 Department of Pediatrics and Communicable Diseases, University of Michigan Medical School, Ann Arbor, MI 48109, USA; 3 School of Chemistry and Molecular Bioscience and Australian Infectious Diseases Research Centre, The University of Queensland, St. Lucia, QLD 4072, Australia; 4 School of Medicine, and Menzies Health Institute Queensland, Griffith University, Parklands, QLD 4222, Australia; 5 Department of Pediatrics, University of California San Diego, La Jolla, CA 92093, USA; 6 Skaggs School of Pharmacy and Pharmaceutical Sciences, University of California San Diego, La Jolla, CA 92093, USA; 7 Department of Molecular Microbiology, School of Medicine, Washington University in St. Louis, St. Louis, MO 63110, USA.

## Abstract

Postpartum women are at increased risk of developing puerperal sepsis caused by group A Streptococcus (GAS). Specific GAS serotypes, including M1 and M28, are more commonly associated with puerperal sepsis. However, the mechanisms of GAS genital tract infection are not well understood. We utilized a murine genital tract carriage model to demonstrate that M1 and M28 GAS colonization triggers TNF-α, IL-1β, and IL-17A production in the female genital tract. GAS-induced IL-17A significantly influences streptococcal carriage and alters local inflammatory responses in two genetically distinct inbred strains of mice. An absence of IL-17A or the IL-1 receptor was associated with reduced neutrophil recruitment to the site of infection; and clearance of GAS was significantly attenuated in IL-17A^−/−^ mice and Rag1^−/−^ mice (that lack mature lymphocytes) but not in mice deficient for the IL-1 receptor. Together, these findings support a role for IL-17A in contributing to the control of streptococcal mucosal colonization and provide new insight into the inflammatory mediators regulating host-pathogen interactions in the female genital tract.

*Streptococcus pyogenes* (group A Streptococcus; GAS) is an obligate human pathogen causing mild active inflammation of the skin and throat, or severe infection when invading sterile sites of the body^1^,^2^. In the 20^th^ century, the advent of antibiotic therapy considerably reduced the burden of GAS disease in Western populations. However, in the last 30 years, there has been a significant re-emergence worldwide of invasive streptococcal diseases associated with the dissemination of hypervirulent clonal strains such as M1T1 GAS^3^,^4^. Investigation of key components and mechanisms of the GAS host-pathogen interaction has therefore become paramount^1^,^2^,^5^.

GAS can penetrate deeper body tissues not only following pharyngeal or epidermal colonization, but also via proliferation in the mucosal tissues of the female urogenital tract. It is recognized as a significant etiologic agent of puerperal sepsis^6^,^7^,^8^. Before advancements in medical hygiene and the availability of antibiotics, puerperal sepsis was a common killer of both mothers and newborns in maternity wards^6^,^9^. With postpartum women at a 20-fold increased risk of disease, puerperal sepsis continues to be a leading cause of maternal mortality^10^; indeed, the incidence of this disease has increased in the last two decades^6^,^11^,^12^,^13^. We recently published a study investigating an outbreak of puerperal sepsis in New South Wales hospitals^14^ that supports the predominant non-random association of GAS serotype M28 with this form of infection^15^,^16^,^17^.

Understanding mucosal immune responses in the female urogenital tract to bacterial pathogens is critical in helping to prevent and treat infections, and may increase awareness of factors promoting premature birth. An important component of mucosal immunity is interleukin-17A (IL-17A), a cytokine activated in response to the presence of bacterial or fungal pathogens that promotes recruitment of phagocytes to the site of infection^18^. While the role of IL-17A has been addressed in response to multiple bacterial and fungal pathogens, there is limited data connecting IL-17A to GAS infections. Work by Cleary and others have begun to show the importance of IL-17A-mediated immunity in response to GAS. Intranasal inoculation of mice with GAS activated IL-17A-producing CD4^+^ Th17 cells from nasal-associated lymphoid tissue; clearance of streptococcal infection was dependent on Th17 lymphocyte polarization in an IL-6 dependent manner^19^. Furthermore, using a murine pharyngitis model it was shown that repeat GAS infections enhanced the migration of GAS-specific IL-17A producing Th17 cells into the brain, increasing the risk of developing autoimmune neurological disorders such as as pediatric autoimmune neurologic disorders associated with streptococcus (PANDAS) and multiple sclerosis^20^. The role of IL-17A in response to GAS urogenital tract infection has not been previously addressed due to lack of a suitable animal model. Although GAS is a strictly human pathogen and its virulence mechanisms are specifically adapted to the interaction with the human immune system, a number of animal models have been developed to mimic certain facets of human GAS infection and investigate the corresponding host immune responses^21^. Recently, murine models of cervico-vaginal colonization suitable for the study of urogenital GAS and group B Streptococcus (GBS) infection have been reported^22^,^23^. To characterize host-pathogen interactions involved in genital tract colonization, we examined the cervico-vaginal colonization potential of a GAS M28 isolate^23^ in parallel with a representative strain of the hypervirulent M1T1 GAS clone^3^,^5^. We find that GAS cervico-vaginal infection triggers host inflammation at the cellular and soluble level, with host IL-17A playing an important role in successful clearance of streptococcal mucosal infection.

## Results

### GAS M28 strain MEW123 and M1T1 strain 5448 establish chronic colonization of the murine female genital tract

**Figure 1:**
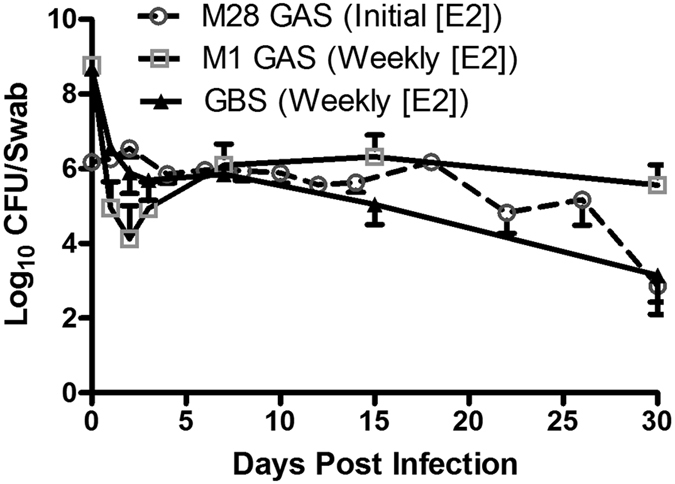
GAS chronically colonizes the female genital tract. Mice were infected with M28 GAS MEW123 or M1 GAS 5448, or GBS 874391 in the vaginal vault and colonization was monitored using colony counts. M28 MEW123 was inoculated in mice treated with estradiol at the onset of the experiment only (Initial [E2]), whereas mice inoculated with M1 GAS 5448 or GBS 874391 received weekly estradiol treatments to maintain estrous phase (Weekly [E2]). Total bacterial numbers and Gram-positive bacteria were also enumerated in colonized animals and showed no significant changes following challenge with GAS or GBS (Supplementary Fig. S1a,b, data not shown). Data are mean ± SEM of 12 mice and represents at least two independent experiments. Statistical comparisons are area under the curve, followed by Mann-Whitney U-test, with significance set at p < 0.05.

We analyzed the fitness of an M28 GAS clinical isolate MEW123^23^ and an invasive M1 GAS strain 5448 for colonization of the genital tract in murine models using two different estrogen-dosing strategies. Estrogen-dosing was provided by: (1) dosing with 0.5 mg estradiol at 48 h prior to and at the time of inoculation (estradiol pulse model), or (2) dosing with 0.1 mg estradiol at 24 h prior to inoculation then once weekly over the course of the experiment to maintain mice in a persistent estrus phase (persistent estrus model). In mice treated with estradiol only at the initiation of infection (estradiol pulse), M28 GAS was recovered from the vaginal vault at levels of 10^5^–10^6^ colony-forming units (CFU)/swab for the first two to three weeks following challenge; colonization persisted for at least 30 days although the M28 GAS strain was eventually cleared from the mucosa (Fig. 1). In the second model in which weekly treatment with estradiol maintained mice in the estrus phase of the estrous cycle, the fitness for colonization of M1 GAS was approximately 1-logfold higher over the time course compared to a strain of GBS (10^6^–10^7^ CFU/swab; Fig. 1; p = 0.0011). The total bacterial counts and levels of Gram-positive bacteria were comparable between these groups (i.e. GAS or GBS colonization; Supplementary Fig. S1a,b).

### Neutrophils dominate cellular infiltrates in response to GAS challenge

**Figure 2:**
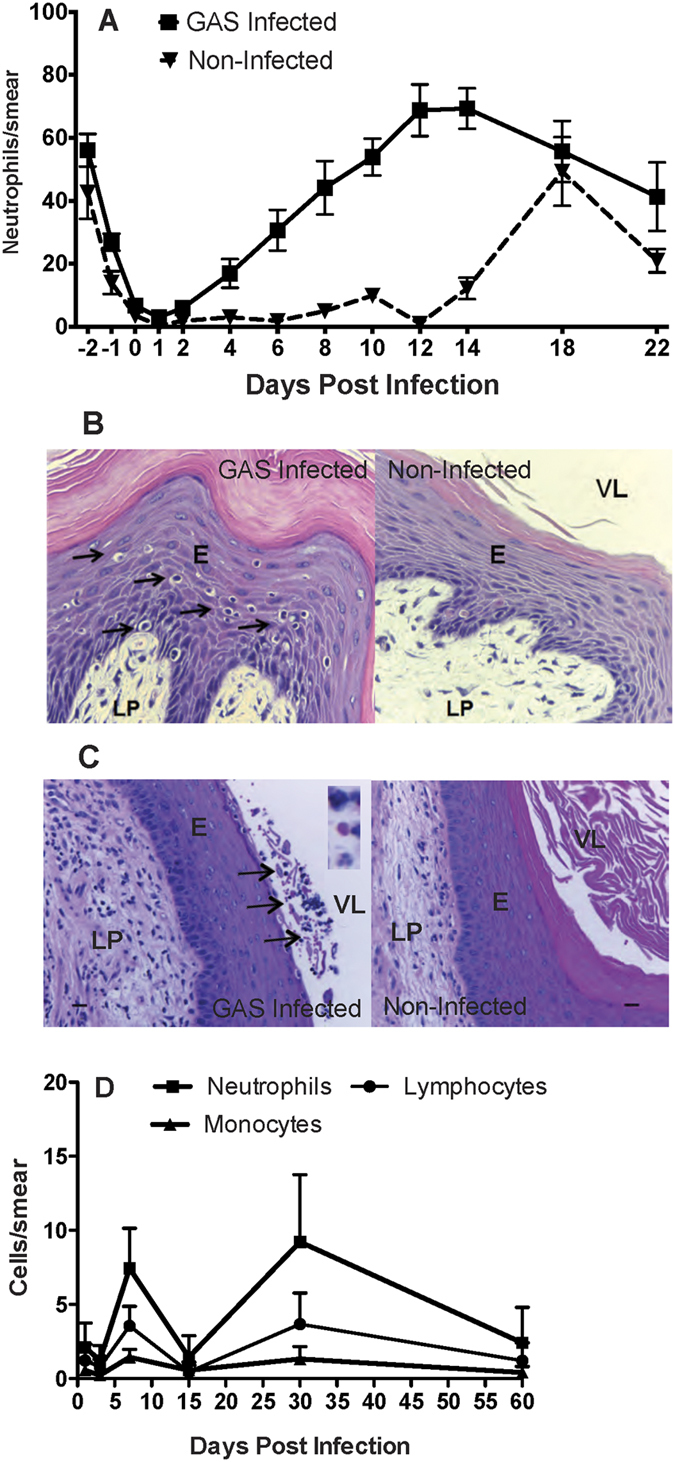
Chronic GAS colonization induces a cellular infiltrate in the female genital tract predominated by neutrophils. (**A**) Neutrophil counts per 100 cells counted in smears of vaginal wash fluid following inoculation of C57BL/6J mice with M28 GAS showing a significant infiltrate compared to non-infected mice (n = 12 mice/point, RM-ANOVA, p < 0.0001). (**B**) The presence of large numbers of leukocytes, including neutrophils, within the epithelial layers of the vaginal mucosa is shown in H&E stained tissue sections collected from mice at day 14 (×40 magnification, E: epithelium, LP: lamina propria, VL: vaginal lumen; arrows indicate increased presence of leukocytes within the epithelium). (**C**) Tissue inflammation in C57BL/6J mice colonized with M1 GAS and maintained in persistent estrous comprised increased numbers of neutrophils (arrows; scale bars are 20 μm, ×20 magnification). (**D**) There was no significant difference in leukocyte numbers in vaginal smears between groups in the persistent estrus model (n = 12 mice/point, data not shown for non-infected group). Data represent at least two independent experiments.

An advantage of the murine vaginal colonization model is the ability to readily interrogate host immune responses to streptococcal colonization, including detection of both cellular migrations into the colonized vaginal mucosa and soluble mediators of inflammation. Prior estrogenization of mice may influence host immune properties in the vaginal mucosa, particularly with regards to inflammatory mediators that normally fluctuate due to the estrous cycle^23^. We compared our pulsed estradiol-dosing scheme versus the weekly dosing scheme in influencing inflammation in response to GAS infection. In the estradiol pulse model, non-infected mice exhibit a low-level of vaginal fluid leukocytes resembling the estrus phase for at least 14 days into the experiment before resuming normal estrous cycling (Fig. 2A). Mice infected with M28 GAS exhibited a significantly increased leukocyte influx, with a heavy neutrophil predominance, beginning as early as 4 days post-infection and peaking at about days 12–14 (p < 0.0001). After 14 days, GAS-infected mice began to resume estrous cycling, making interpretation of leukocyte levels after this time point difficult (Fig. 2A). Examination of H&E-stained sections of vaginal tissue demonstrated that M28 GAS induced notable cellular infiltrates including intraepithelial neutrophils within the vaginal lumen when compared to non-infected mice. There was also a mild increase in sub epithelial tissue inflammation, compared with non-infected mice (Fig. 2B).

We utilized the persistent estrus model to assess cellular infiltrates over a longer time course following GAS infection. Mice treated using this model exhibited a mixed inflammatory cell infiltrate (comprised mainly of neutrophils) in the mucosa, smooth muscles and adjacent adipose tissue with either moderate or severe inflammation (Fig. 2C); observations largely consistent with the results observed in the estradiol pulse model. Occasional intra-epithelial abscesses were observed, and bacteria were noted in the vaginal lumen mixed with necrotic debris. Vaginal smears, used to assess the lumen infiltrate, showed no significant differences between the numbers of leukocytes in mice colonized with GAS compared to non-infected controls (Fig. 2D, data not shown). Thus, similar to M28 GAS in the estradiol pulse model, M1 GAS induces an inflammatory cell infiltrate in the vaginal tissue in the persistent estrus model that is dominated by neutrophils. Cellular infiltrates induced by GAS in the vaginal lumen, however, are suppressed by hormone treatment in a model of continued estrus.

### GAS induces inflammatory mediators including IL-17A in the female genital tract

**Figure 3:**
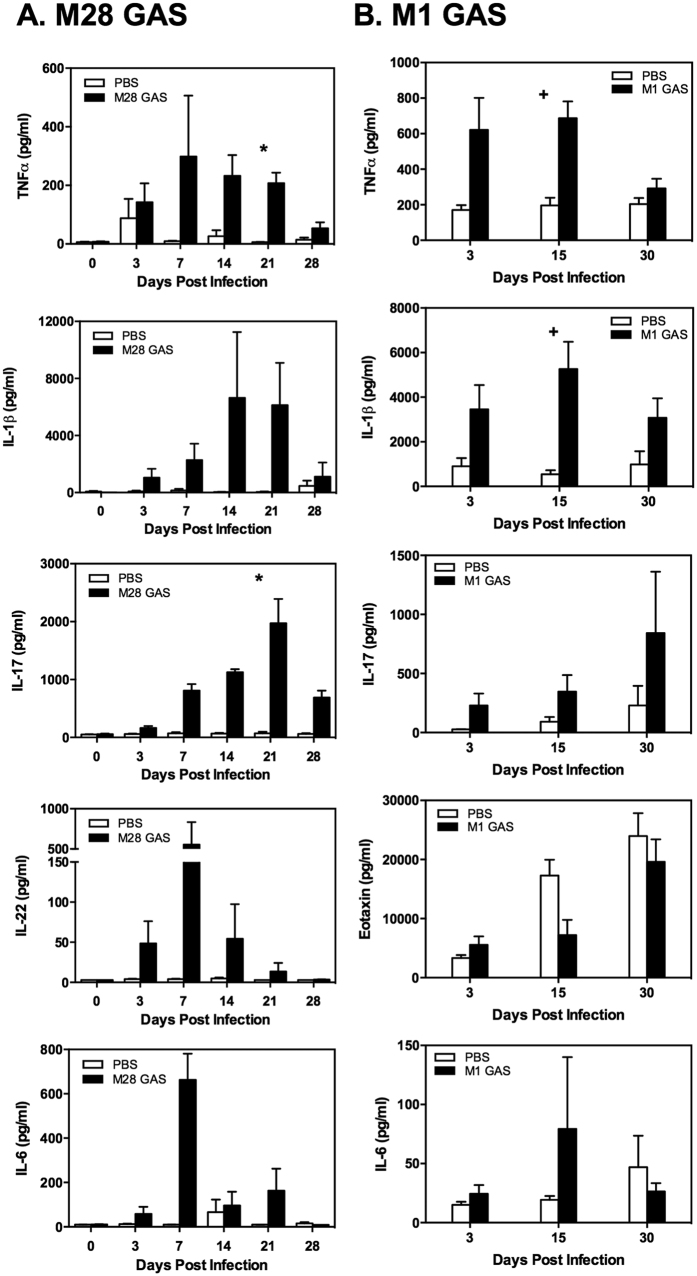
GAS induces the secretion of inflammatory mediators including IL-17A in murine cervico-vaginal tissue. Mice were infected with either (**A**) M28 GAS or (**B**) M1 GAS and multiplex protein assays were performed on homogenized tissues collected at various time points post-infection. Sustained levels of IL-1β, TNFα and IL-17A were detected in the host response to both GAS strains. Data are mean ± SEM, with at least 8 mice/group. Significance was analyzed using a Kruskal-Wallis test, followed by a Dunn’s multiple comparison post-test using a p value of  < 0.05. +: p < 0.01; *p < 0.05.

Multiplex protein assays were performed on cervical/vaginal tissues collected at days 3 to 30 post-infection for M28 and M1 GAS and compared with non-infected (PBS) controls. There was a pattern of inflammatory cytokine expression detected in mice inoculated with M28 GAS, with significant increases in IL-1β, IL-17A, and TNFα, and substantial increases in IL-6 and IL-22 (Fig 3A). The responses of mice to M1 GAS infection in the persistent estrus model, used to minimize changes in inflammatory conditions due to normal hormonal cycling, were largely consistent with those to M28 GAS infection; only few analytes had significant changes compared to controls (Fig. 3B) including IL-1β (higher in infected mice at days 3, 15 (p < 0.01) and 30, TNF-α (higher in infected mice at day 3 and 15 (p < 0.01)), respectively) and eotaxin (lower in infected mice at day 15). The levels of IL-17A were elevated above controls 7-fold at day 3, 3-fold at day 15 and 4-fold at day 30. Additional insignificant analytes for M1 GAS colonization are shown in Supplementary Fig. S2.

### Vaginal IL-17A contributes to the control of GAS chronic colonization and promotes local cellular infiltration

**Figure 4:**
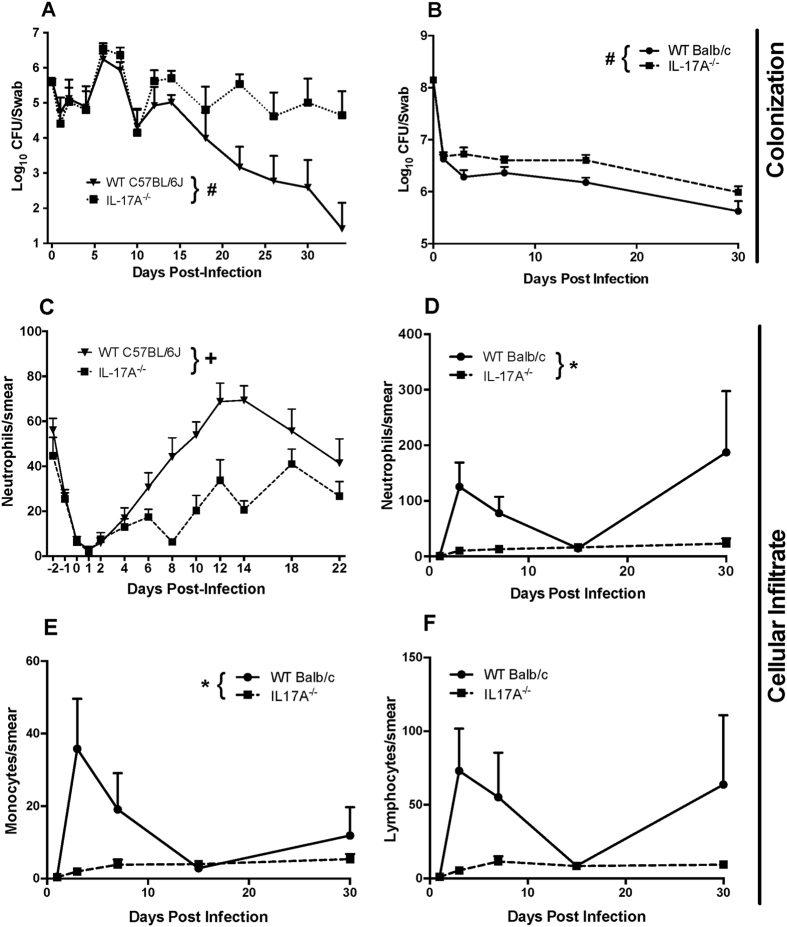
IL-17A contributes to control of GAS colonization. CFU levels of M28 GAS (**A**) and M1 GAS (**B**) in C57BL/6J and Balb/c models, respectively. IL-17A^−/−^ mice of either the C57BL/6J (**A**) or Balb/c (**B**) genetic backgrounds exhibited significantly greater GAS CFU counts in vaginal specimens compared to WT C57BL/6J mice (n = 12 mice/group, RM-ANOVA, ^#^p < 0.01) or Balb/c mice (n = 20 mice/group, area under the curve analysis, followed by Mann-Whitney U test, ^#^p < 0.01). IL-17A^−/−^ mice also exhibited an attenuated neutrophil influx in colonized vaginal washes compared to C57BL/6J control mice (panel **C**, n = 12 mice/group, RM-ANOVA, +: p < 0.001). Panels (**D–F**) represent cell counts of smears from vaginal swabs of Balb/c mice showing neutrophils (**D**), monocytes (**E**), and lymphocytes (**F**) and demonstrate significantly greater levels of neutrophils and monocytes at day 3 post-infection. Data are mean ± SEM of 9–15 mice and are 2 separate experiments combined. Mann-Whitney U-tests for each time point were used to compare inflammatory cell counts, with significance set at p < 0.05 for both, *p < 0.05.

Given sustained IL-17A responses of mice to M28 and M1 GAS following vaginal colonization in two estrogenization models, and recognizing emerging evidence that IL-17A play a role in GAS infection^19^,^20^ we tested the role of IL-17A in GAS genital tract colonization. IL-17A knockout (KO) mice (C57BL/6J background) were infected with M28 GAS and had significantly higher GAS vaginal fluid CFU counts over time compared to wild-type (WT) controls (Fig. 4A). In this model, WT mice harbored an average of 99% fewer GAS in vaginal swabs after 3 weeks of infection compared to IL-17A^−/−^ mice (1–5 × 10^5^ CFU/swab versus 10^2^–10^3^ CFU/swab; p < 0.01). IL-17A^−/−^ mice on a Balb/c background, infected with M1 GAS using the persistent estrus model, also exhibited significantly higher GAS colonization over time compared to WT mice (4–7 × 10^6^ CFU/swab versus 2–4 × 10^6^ CFU/swab; p = 0.0073) (Fig. 4B). Thus, IL-17A-deficient mice are attenuated in their ability to clear GAS compared with WT mice, establishing that IL-17A contributes to controlling GAS levels in the female genital tract.

**Figure 5:**
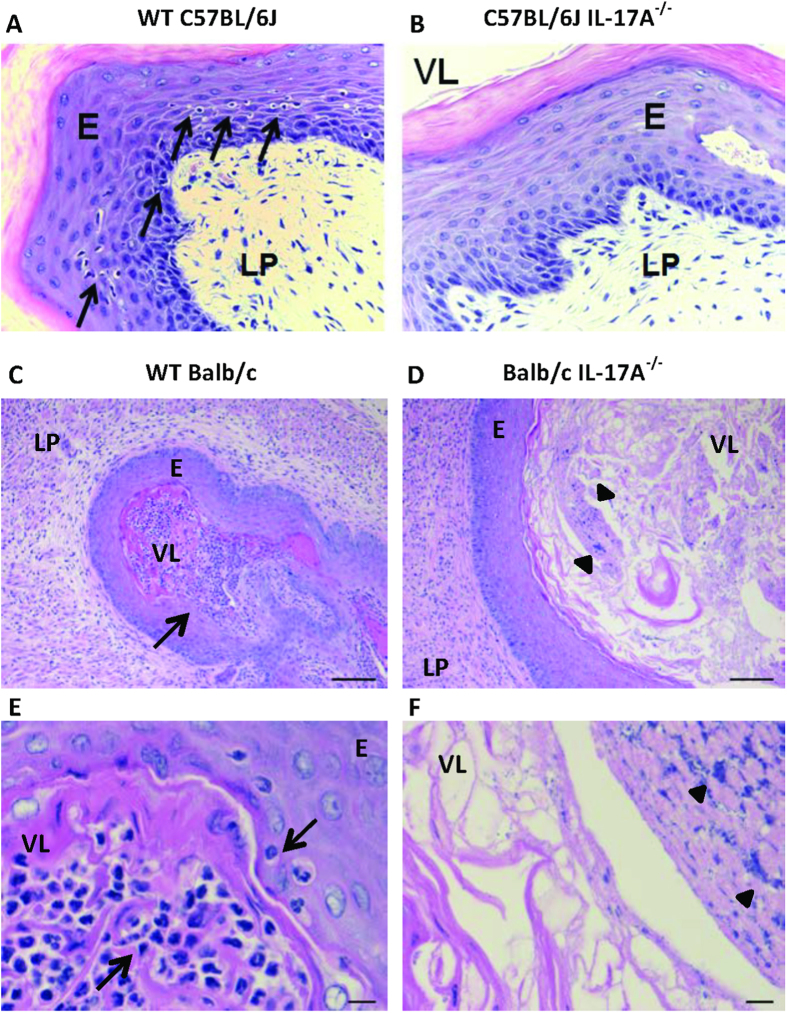
H&E stained sections of vaginal tissue in C57BL/6J and Balb/c mice highlight differences in inflammation between WT and IL-17A^−/−^ animals. M28 GAS colonized C57BL/6J mice (**A**) and IL-17A^−/−^ mice (**B**) showing vaginal tissue at day 14 post-infection, VL = vaginal lumen, E = epithelium, LP = lamina propria, arrows indicate inflammatory cell presence, ×40 magnification. M1 GAS colonized Balb/c (**C**,**E**) and IL17A^−/−^ (**D**,**F**) vaginal tissue at day 30 post-infection, arrows indicate inflammatory cells and arrow heads highlight bacterial presence. Scale bars are 100 μm for ×10 magnification (panels **C**,**D**) and 5 μm for ×63 magnification (panels **E**,**F**).

Neutrophil counts in vaginal washes from C57BL/6J IL-17A^−/−^ mice infected with M28 GAS were significantly lower compared with WT mice (p < 0.001) (Fig. 4C). In the persistent estrus model in Balb/c mice infected with M1 GAS, IL-17A^−/−^ mice also exhibited significantly lower levels of neutrophil (Fig. 4D) and monocyte (Fig. 4E) infiltration at 3 days post-infection (p = 0.0129 and 0.0276, respectively). Lymphocyte infiltration was also lower in IL-17A^−/−^ Balb/c mice compared with WT mice of the Balb/c background at 3 days post-infection, but this did not reach statistical significance (Fig. 4F). Histopathology analysis of H&E sections prepared at day 14 post-infection demonstrated a modest increase in inflammation in the vaginal lumen and epithelium of WT mice infected with M28 GAS compared with IL-17A^−/−^ mice (Fig. 5A,B, arrows). We also observed histopathologic evidence of greater levels of inflammation in WT mice versus IL-17A^−/−^ in the luminal and sub epithelial stroma of vaginal mucosa at day 30 in the Balb/c persistent estrus model (Fig. 5C–F; arrows), at which time bacteria were still present in the lumen of IL-17A^−/−^ mice (Fig. 5D,F; arrowheads). The consistency of these findings in both models establishes that IL-17A contributes to the local cellular inflammatory response during chronic GAS colonization of the female genital tract.

### Mature lymphocytes and IL-17A promote streptococcal mucosal clearance independent of leukocyte recruiting activity

**Figure 6:**
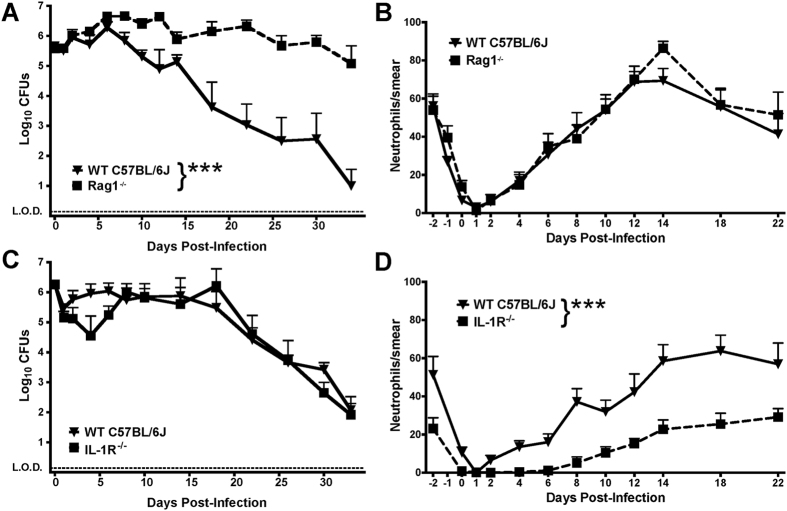
Rag1^−/−^ mice, but not IL-1R^−/−^ mice, are attenuated for clearance of M28 GAS vaginal colonization. Panels (**A,C**) show CFU counts recovered from vaginal washes of Rag1^−/−^ mice and IL-1R^−/−^ mice, respectively, each infected with 10^6^ CFU GAS M28 strain MEW123. Rag1^−/−^ mice show significantly greater CFU counts from vaginal washes isolated serially over time compared with WT C57BL/6J mice (n = 8 mice/point, RM-ANOVA, ***p < 0.001). IL-1R^−/−^ mice did not differ significantly from WT controls in their ability to clear carriage (n = 10 mice/point). Panels (**B,D**) show neutrophil counts per 100 cells counted in smears from vaginal washes of GAS MEW123 infected Rag1^−/−^ mice and IL-1R^−/−^ mice, respectively. No significant difference in neutrophil recruitment was found between Rag1^−/−^ mice and WT mice (**B**, n = 8 mice/point, RM-ANOVA, p = 0.224). Mice lacking the IL-1 receptor were significantly attenuated for neutrophil recruitment compared to WT mice (**D**, n = 8 mice/point, RM-ANOVA,***p < 0.001).

A major source of IL-17A is mature CD4^+^ T lymphocytes, primarily those of the Th17 class. Alternative sources of IL-17A include neutrophils, γδ T cells, and NKT cells^18^. To investigate whether clearance of GAS vaginal mucosal colonization requires IL-17A-producing CD4^+^ Th17 lymphocytes, we tested M28 GAS colonization in C57BL/6J mice lacking the Recombination Activating Gene 1 (Rag1), which do not produce mature T or B lymphocytes. Streptococcal clearance from Rag1^−/−^ mice was significantly attenuated compared with WT mice, with significantly greater quantities of GAS recovered from vaginal washes of Rag1^−/−^ mice over time (Fig. 6A, p < 0.001). Despite increased GAS vaginal carriage, the Rag1^−/−^ mice did not appear toxic or ill over the course of the experiment. In contrast to IL-17A^−/−^ mice, Rag1^−/−^ mice were not deficient in neutrophil recruitment to GAS infected vaginal tissue when compared with WT mice controls (Fig. 6B), suggesting non-lymphocyte sources of IL-17A or other pro-inflammatory cytokines were still active. Histopathology analysis of H&E sections prepared at day 14 post-infection demonstrated an equal or greater degree of inflammation in the vaginal lumen and epithelium of Rag1^−/−^ mice infected with M28 GAS compared with WT mice controls (Supplementary Fig. S3a). Furthermore, cytokine levels from vaginal washes of M28 GAS infected Rag1^−/−^ and WT mice demonstrated that the Rag1^−/−^ mice produced greater quantities of IL-1β, IL-6, and TNFα at 14, 21, and 28 days post-infection compared to WT mice (Supplementary Fig. S3b). In contrast, WT mice produced significantly more IL-17A compared with Rag1^−/−^ mice, supporting the conclusion that mature lymphocyte sources of IL-17A are responsible for the majority of IL-17A expression in response to GAS infection in this model. An exaggerated innate immune response in Rag1^−/−^ mice due to the lack of regulatory T cells has been described by other investigators and may explain the apparently normal neutrophil response to infection observed in the Rag1^−/−^ mice^24^.

Based on the significant induction of IL-1β observed in these models (Fig. 3A,B), we next assessed GAS clearance in mice lacking the IL-1 receptor (IL-1R^−/−^) to determine if IL-1β signaling was necessary for clearance and/or neutrophil recruitment. Importantly, mice deficient in IL-1 signaling were not significantly different from WT controls in their ability to clear the M28 GAS vaginal carriage (Fig. 6C). Neutrophil recruitment to vaginal mucosa in the IL-1R^−/−^ mice was significantly attenuated compared to WT controls, with detection of neutrophils observed in vaginal smears only after one week post-infection (Fig. 6D, p < 0.001). Overall, the lack of clearance in Rag1^−/−^ and IL-17A^−/−^ mice, but not IL-1R^−/−^ mice, supports the role IL-17A and adaptive immunity through mature lymphocytes in promoting clearance of GAS vaginal mucosal infection and carriage.

## Discussion

Historically, GAS has been a major causative agent of puerperal sepsis and even today remains a significant cause of postpartum wound infections. The reasons why women are more susceptible to GAS infection in the postpartum period have not been studied in detail although speculation exists that rapid changes in hormone levels at the time of delivery may impair mucosal immunity to GAS among other pathogens^25^. The strains most commonly associated with the development of puerperal sepsis belong to the M1 and M28 serotypes, accounting for 73% of severe cases, with M28 strains being associated with a disproportionally higher rate of asymptomatic vaginal colonization^26^. While GAS puerperal sepsis is most frequently linked to M28 strains, highest mortality is linked with M1 and M3 (*emm1* and *emm3*) isolates^27^. Human female vaginal carriage rates for GAS are low (0.03–1%)^28^,^29^ compared to vaginal colonization by GBS, which ranges from 10–30%. To model streptococcal vaginal carriage we have utilized a murine vaginal carriage model for GAS and GBS that have previously been validated as achieving sustained colonization and carriage of these bacteria for investigation of host-pathogen interactions influencing infections in this niche^22^,^23^. This study adds to these investigations of GAS vaginal mucosal infection establishing that representative strains of GAS M1 and M28 serotypes are capable of colonizing the murine vaginal mucosa at levels comparable to, if not greater, than serotype III GBS 874391.

We found that both M1 and M28 GAS induce elevations in host inflammatory markers in the genital tract including the acute inflammatory cytokines, IL-1β and TNF-α. These were increased throughout infection in a manner comparable to findings in the acute phase in a murine model of GAS induced arthritis^30^. We observed significant increases in cellular infiltrate in the lumen of the genital tract in the persistent estrus model of M1 GAS infection, but only in neutrophils and monocytes at day 3. In contrast, M28 GAS elicited significant cytokine increases in TNF-α, IL-17A, IL-6, IL-1β, and IL-22, as early as 3 to 7 days post-infection in the estradiol pulse model, and a significant cellular infiltrate in the lumen was observed in colonized WT mice compared to non-infected or IL-17A^−/−^ mice. In previous work we demonstrated GAS vaginally-colonized Balb/c mice show more cellular inflammation in the vaginal lumen compared with colonized C57BL/6J mice, confirming that direct comparisons between different mouse strains cannot always be made^23^. In addition, different estradiol-dosing protocols (i.e., initial versus weekly) may account for some differences in host responses noted between these two experimental approaches. However, we believe these differences in outcomes with strain variability or estradiol-dosing can be exploited to highlight different aspects of streptococcal carriage and the host immune response depending on the question under investigation; we believe the flexibility in experimental design provides a strength to these models for future experiments of vaginal mucosal host-pathogen interactions and investigation of persistent mucosal carriage.

Levels of IL-17A were increased following infection in both Balb/c and C57BL/6J mice, and this was associated with the host response to both M1 and M28 GAS. Chronic arthritis in mice, induced by exposure to GAS cell wall fragments, is driven by IL-17A which is increased in addition to IL-23, another Th17 cytokine^30^, while in acute sepsis IL-17A gene expression has been shown to have more sustained upregulation^31^.This combined with increases in IL-1β and IL-6, is indicative of an active inflammatory response. In this study, we determined the role that IL-17A plays in GAS colonization by comparing WT mice and mice deficient in IL-17A. GAS was recovered at significantly higher levels in Balb/c IL-17A^−/−^ animals during the first two weeks after challenge converging to WT levels at day 30, indicating a significant role of IL-17A in the host response to GAS colonization. Similar results were obtained with C57BL/6J mice deficient in IL-17A in the context of M28 GAS colonization and has previously been noted in a murine model of *N. gonorrhoeae* infection in which IL-17A^−/−^ mice exhibited prolonged genital tract infection. Importantly, prior studies have demonstrated that IL-17A^−/−^ mice generated in C57BL/6J or Balb/c backgrounds have essentially normal immune cell populations in the thymus, lymph nodes, and spleen, suggesting the observed defects in bacterial clearance are not simply due to major differences in leukocyte cellular reserves, but rather due to specific impairment of leukocyte recruitment or other effector activities of IL-17A itself 32,33. Likewise, the circulating leukocyte pool of WT and IL-1R KO mice is similar with no major cell type deficiencies^34^,^35^. Overall, this suggests that studies utilizing mice strains lacking expression of IL-17A or the IL-1R can adequately serve as model systems to address the role of these important cytokines in coordinating host responses to infection.

Mucosal infection with an M1 GAS strain induced a strong antigen-specific Th17 response in cells isolated from nasal-associated lymphoid tissue of C57BL/6 mice, with CD4^+^ cells producing IL-17A, whereas intravenous and subcutaneous infection produced IFN-γ secreting cells^19^. This response was found to be IL-6-dependent. More recently, a distinctive immune response involving increased production of IL-17A within the vaginal tract of CD1 mice persistently colonized with GBS was also reported. Treatment of the mice with recombinant IL-17 was shown to enhance clearance of GBS^36^. In addition, lack of IL-17A was shown to delay the influx of neutrophils to the site of gonococcal vaginal infection, indicating a suppression of the innate immune response^37^. In our study, examination of vaginal smears also revealed a significant increase in inflammation in WT Balb/c mice in the first week of infection, while infiltrates remained low in IL-17A^−/−^ mice in the same period. Thus, our findings are consistent with a host protective role for IL-17A and suggest a model in which IL-17A mediated inflammation is critical for the eradication of streptococci from the female genital tract mucosa. In a number of infections of various etiologies at the mucosal surface, IL-17A has a crucial role in the recruitment of neutrophils and lymphocytes and activation of antimicrobial peptide defenses^38^. More broadly, these observations point to a key role of IL-17A in inflammatory signaling in the genital tract. However, it is likely that multiple other factors in addition to IL-17 contribute to the control of GAS colonization at mucosal surfaces given the complexities in host responses that mediate antimicrobial effector mechanisms at these sites^32^,^39^.

Interestingly, Rag1^−/−^ mice did not exhibit a defect in neutrophil recruitment in response to GAS infection compared to WT mice. This may suggest that in this acute setting non-lymphocyte sources of IL-17A (e.g., neutrophils, epithelial cells) or other inflammatory mediators participate in promoting recruitment of neutrophils to the infected vaginal epithelium^40^. In support of this hypothesis we found that both IL-17A and IL-1β signaling were important to promote neutrophil recruitment to GAS infected vaginal mucosa. Neutrophils are important mediators of various antimicrobial defense mechanisms at the mucosa including acute inflammation, microbial killing via the NADPH oxidase complex, and depletion of local oxygen that can stabilize hypoxia-inducible factor and promote the maintenance and restoration of mucosal barrier function^39^. However, we observed that only Rag1^−/−^ mice lacking mature lymphocytes or IL-17A^−/−^ mice were deficient in GAS clearance. Adoptive transfer of CD4^+^/IL-17A^+^ T cells from previously GAS infected animals into naïve animals have proven to be effective at providing protection against intranasal GAS infection^41^, supporting the role of both IL-17A and T cells in GAS clearance from the mucosa. In our experiments, mice lacking the IL-1 receptor were attenuated for neutrophil recruitment, especially in the first week of infection; a moderate recruitment of neutrophils after this first week may be due to lymphocyte recruitment and production of IL-17A, possibly explaining the ability of the IL-1R^−/−^ mice to ultimately clear GAS carriage appropriately. We appreciated in the murine vaginal carriage model time points in which high CFU counts of GAS were continuously recovered despite the presence of a significant neutrophil response in the vaginal lumen (as seen in Rag1^−/−^ mice, Fig. 6A,B), suggesting that neutrophils alone may not be completely sufficient to eradicate mucosal carriage in this compartment. A possible explanation for this discrepancy may be that Rag1^−/−^ mice would be predicted to not make sufficient opsonizing antibody given their lack of mature B cells. Opsonization is a major mechanism of clearance of GAS, at least in blood, but perhaps also at the mucosa^1^; GAS counters the opsonization process with hyaluronate capsule and expression of M protein and other factors which resist opsonization and phagocytic ingestion^42^. In the absence of opsonizing antibody the neutrophils are present in significant numbers but seem to be unable to effectively clear GAS in the Rag1^−/−^ mice. This impairment is likely further compounded by properties of neutrophils promoting resolution of inflammation at sites of mucosal infection or injury. There is accumulating evidence that neutrophil accumulation at sites of mucosal infection along with neutrophil-mediated consumption of local available oxygen via the NADPH complex may further downregulate inflammatory mediator expression by epithelial hypoxia inducible factor, promoting a dampening or resolution of mucosal inflammation^39^. Limited activation of neutrophils in the female reproductive tract may also be a general feature aiding in tolerance to developing fetus^25^. Furthermore, the high doses of estradiol required to achieve long-term carriage in these murine models may also be somewhat influencing activation of the host immune response even to high-levels of GAS present; this is a limitation of this model that must be taken into consideration when interpreting these results. As such, a combination of these features may explain why GAS can persist in the setting of high concentrations of vaginal neutrophils observed in the Rag1^−/−^ mice in this model.

The susceptibility to sepsis caused by Gram-positive bacteria and increased 28-day mortality in severe sepsis and septic shock patients has been linked to polymorphisms in the IL-17A gene^43^. This has implications for patients at risk of puerperal sepsis and further supports our findings of IL-17A contributing to controlling genital tract GAS colonization and risk of puerperal sepsis development.

Here we have demonstrated that the two most common GAS serotypes causing puerperal sepsis are able to colonize the murine female genital tract. The M1 and M28 strain both triggered release of significant amounts of acute inflammatory cytokines, IL-1β, TNF-α and IL-17A, all of which have been linked to GAS disease pathogenesis. We have also demonstrated that IL-17A contributes to controlling GAS levels in the genital tract and influences local inflammatory cytokine activity and cellular infiltration, which we conclude promotes GAS clearance. It is possible however that the findings of the current study may not be equally applicable to all GAS strains. Nonetheless, this model provides critical new insight into both the bacterial virulence and host immune factors that affect the ability of this organism to colonize the female genital tract and contribute to puerperal sepsis.

## Methods

### Bacterial Strains

The GAS isolates used in this study were MEW123, a streptomycin-resistant derivative of an M28 clinical throat isolate^23^; 5448, a representative strain of the globally disseminated M1T1 clone^44^. Bacterial strains were routinely cultured on horse-blood agar (Biomerieux, Baulkham Hills, NSW, Australia) or in Todd-Hewitt (Difco Laboratories, North Ryde, NSW, Australia) supplemented with 1% (w/v) yeast extract (THY) broth, at 37 °C without shaking. Mutants were grown on THY agar supplemented with 100 μg/mL Sp. The hyper-virulent ST-17 type III GBS strain 874391^45^ was used in several comparative assays as previously described^22^.

### Murine genital tract infections and infectious load monitoring

Animal experiments using C57BL/6J mice were performed at Washington University, and the University of Michigan Schools of Medicine, and Griffith University. The methods used in all animal studies were carried out in accordance with the approved guidelines of Washington University, and the University of Michigan Schools of Medicine, and Griffith University. All experimental protocols for animal experiments were approved by the Institutional Animal Care and Use Committees of Washington University, the University of Michigan, and the Griffith University Animal Ethics Committee (approvals: UM PRO5073, MSC/03/12/AEC). Female 6–8 week old C57BL/6J (#000664), IL-1 receptor KO mice (IL-1R^−/−^ #003245), and Rag1 KO Mice (Rag1^−/−^ #002216) mice were purchased from The Jackson Laboratory, Bar Harbor, ME, and The Animal Resource Centre, Western Australia. Female 6–8 week old IL-17A^−/−^ of the C57BL/6 background were originally obtained from Yoichiro Iwakura (Tokyo University of Science, Japan) via Bethany Moore (University of Michigan) and bred in the laboratory of J.B.W. Experiments using 6–8 week old Balb/c mice and an IL-17A^−/−^ derivative, which was acquired from Yoichiro Iwakura (Tokyo University of Science, Japan)^33^ and bred in the laboratory of G.C.U. were performed at Griffith University. The vaginal colonization protocols used in this study were as previously reported^23^,^22^. Mice received either (i) intraperitoneal injections of 0.5 mg 17-β-estradiol (Sigma Aldrich) in sterile sesame oil (Sigma Aldrich), once at 48 h prior to inoculation and again on the day of inoculation (Day 0)^23^, or (ii) subcutaneous injections of 0.1 mg 17β-estradiol in castor oil once at 24 h prior to inoculation and weekly thereafter to maintain them in the estrus stage of the estrous cycle^22^. The former protocol maintained mice in the estrous phase for a minimum of 14 days before they may regain estrous cycling.

On Day 0, mice were inoculated with either (i) ~1 × 10^6^ CFU GAS strain M28 MEW123 in 20 μl of PBS or (ii) ~1 × 10^8^ CFU of GAS strain M1 5448 or GBS in 10 μL of PBS. At intervals following inoculation the vaginal vault was either (i) washed with 50 μl of sterile PBS; washes were diluted in PBS and subsequently plated onto THY agar supplemented with streptomycin (1 mg/ml) for viable counts^23^, or (ii) sampled using cervico-vaginal swabs (Copan, Murrieta, CA) as previously described^22^. Swabs were cultured on 5% horse-blood tryptic soy agar (TSA, Oxoid, Adelaide, SA, Australia) for viable counts of total bacteria, and 5% horse-blood Columbia agar (ColNAC; Oxoid) with 15 μg/mL nalidixic acid and 10 μg/mL colistin (Sigma-Aldrich, Castle Hill, NSW, Australia) for viable counts of Gram-positive bacteria. To enumerate GAS or GBS in the swabs the CHROMagar StrepB (selective for GBS; Micromedia, Moe, VIC, Australia) formulation was modified by Micromedia to exclude the addition of Bacitracin, allowing differentiation of GAS and GBS.

### Cytological and histological assessment of vaginal cellular inflammatory infiltrates

Upon collection of cervico-vaginal swabs, smears on glass slides were generated to examine the cellular infiltrate into the cervico-vaginal lumen. Slides were air dried and fixed in methanol for 30 sec before staining with Quick Dip Differential Cell Stain (Thermo Fisher Scientific, Scoresby, VIC, Australia). Slides were scanned using an Aperio Scanscope (Leica, North Ryde, NSW, Australia) at 40x magnification. Images were examined to enumerate lymphocytes, neutrophils and monocytes in those mice that were in the estrus stage of the estrous cycle only. This was done to avoid biasing infiltrate counts due to estrous stages where leukocytes are naturally dominant. Enumeration of the inflammatory cellular infiltrate was performed in a non-blinded manner; however random smears were selected and examined in a blinded method by a third party to confirm the accuracy of the results. Cervico-vaginal tissues were collected at indicated time points post-infection, fixed in 10% buffered-formalin and processed for hematoxylin and eosin (H&E) staining. Imaging was performed using an Axio Imager.M2 microscope (Carl Zeiss MicroImaging GmbH, Germany). In all microscopy studies, the investigator(s) reading the slides were experienced in cellular morphology and histology of murine vaginal epithelium and were blinded to the specimen source. Multiple fields were imaged from each slide specimen to obtain accurate cell counts and to obtain representative images shown in the figures.

### Measurement of soluble inflammatory cytokines and chemokines

17β-estradiol primed mice were infected with either the 5448 M1 isolate or sham infected with 10 μL sterile PBS as mentioned above, and received weekly 17β-estradiol injections. Animals were euthanized on days 3, 15 and 30 post-infection and the cervico-vaginal regions were removed and each collected into 150 μL protease inhibitor cocktail (Roche, Castle Hill, NSW, Australia). Tissues were homogenized and clarified as previously described^22^; supernatants were stored at −80 °C until use. Fifty microliters of samples were used in a 23-plex multiplex protein assay according to manufacturer’s instructions (Bio-Rad, Gladesville, NSW, Australia). Similar experiments were performed in C57BL/6J mice vaginally colonized with GAS MEW123 or sham inoculated with sterile PBS. Animals were euthanized on days 3, 7, 14, 21, and 28 post-infection, the cervico-vaginal regions were removed and homogenized, and processed as above. Samples were assayed using a custom magnetic bead-coupled multiplex protein assay according to manufacturer’s instructions (EMD Millipore, Billerica, MA, USA).

### Statistics

The levels of colonization between the groups were compared over time using area under the curve analyses, followed by a Mann-Whitney U-test, with significance set for p < 0.05. Where indicated a repeated measures analysis of variance (RM-ANOVA) assay was used to compare CFU recovery or leukocyte counts from vaginal washes from serial specimens over time. For multiplex protein arrays and inflammatory cellular infiltrates a Kruskal-Wallis test, followed by a Dunn’s multiple comparison post-test (multiple comparisons) were used with significance set at p < 0.05. GraphPad Prism (version 5.04) software was used for all statistical analyses. Numbers of mice and replicates used in experiments is indicated in figure legends.

## Additional Information

**How to cite this article**: Carey, A. J. *et al.* Interleukin-17A Contributes to the Control of *Streptococcus pyogenes* Colonization and Inflammation of the Female Genital Tract. *Sci. Rep.* **6**, 26836; doi: 10.1038/srep26836 (2016).

## Supplementary Material

Supplementary Information
