# Supplementary material for: Interleukin-17A Contributes to the Control of Streptococcus pyogenes Colonization and Inflammation of the Female Genital Tract
Source: Sci Rep. 2016 May 31;6:26836. doi: 10.1038/srep26836 (PMC4886215; doi:10.1038/srep26836)
Supplement: Supplementary Information [file srep26836-s1.doc]

**Supplementary Data**

**Interleukin-17A Controls Streptococcus pyogenes Colonization and Inflammation of the Female Genital Tract**

Alison J. Carey^1^, Jason B Weinberg^2^, Suzanne R. Dawid^2^, Carola Venturini^3^, Alfred K. Lam^4^, Victor Nizet^5^, Michael Caparon^6^, Mark J. Walker^3^, Michael E. Watson^2^, and Glen C. Ulett^1^

^1^School of Medical Sciences, Menzies Health Institute of Queensland, Griffith University, Parklands, QLD, Australia 4222; ^2^Department of Pediatrics and Communicable Diseases, University of Michigan Medical School, Ann Arbor, MI 48109; ^3^School of Chemistry and Molecular Bioscience and Australian Infectious Diseases Research Centre, The University of Queensland, St. Lucia, QLD, Australia 4072. ^4^School of Medicine, Menzies Health Institute of Queensland, Griffith University, Parklands, QLD, Australia 4222; ^5^Department of Pediatrics, University of California San Diego, La Jolla, CA 92093 and Skaggs School of Pharmacy and Pharmaceutical Sciences, University of California San Diego, La Jolla, CA 92093; ^6^Department of Molecular Microbiology, School of Medicine, Washington University in St. Louis, St. Louis, MO 63110.


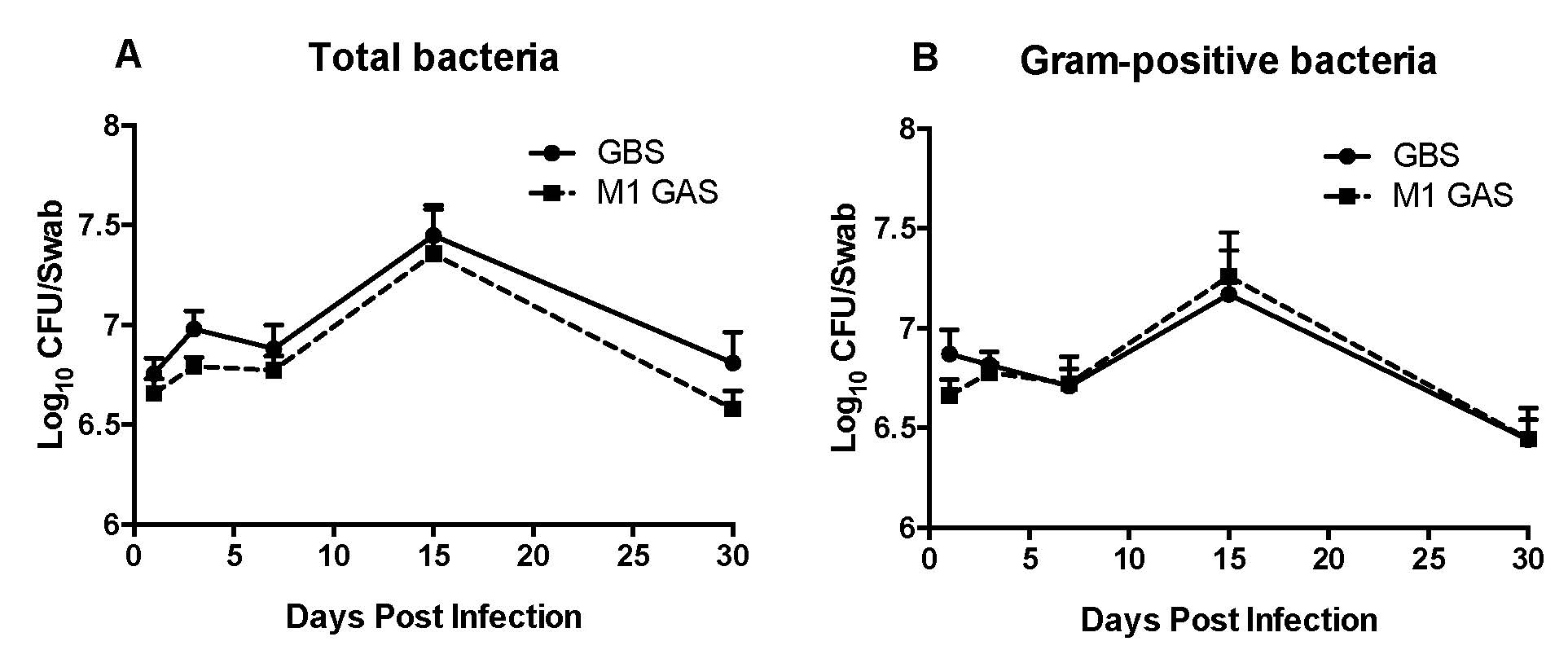


**Supplemental Figure 1:** The levels of total and Gram-positive bacteria from GBS and M1 GAS colonized animals. Data is mean ± standard error of the mean of 12 mice and represent two individual experiments.

**
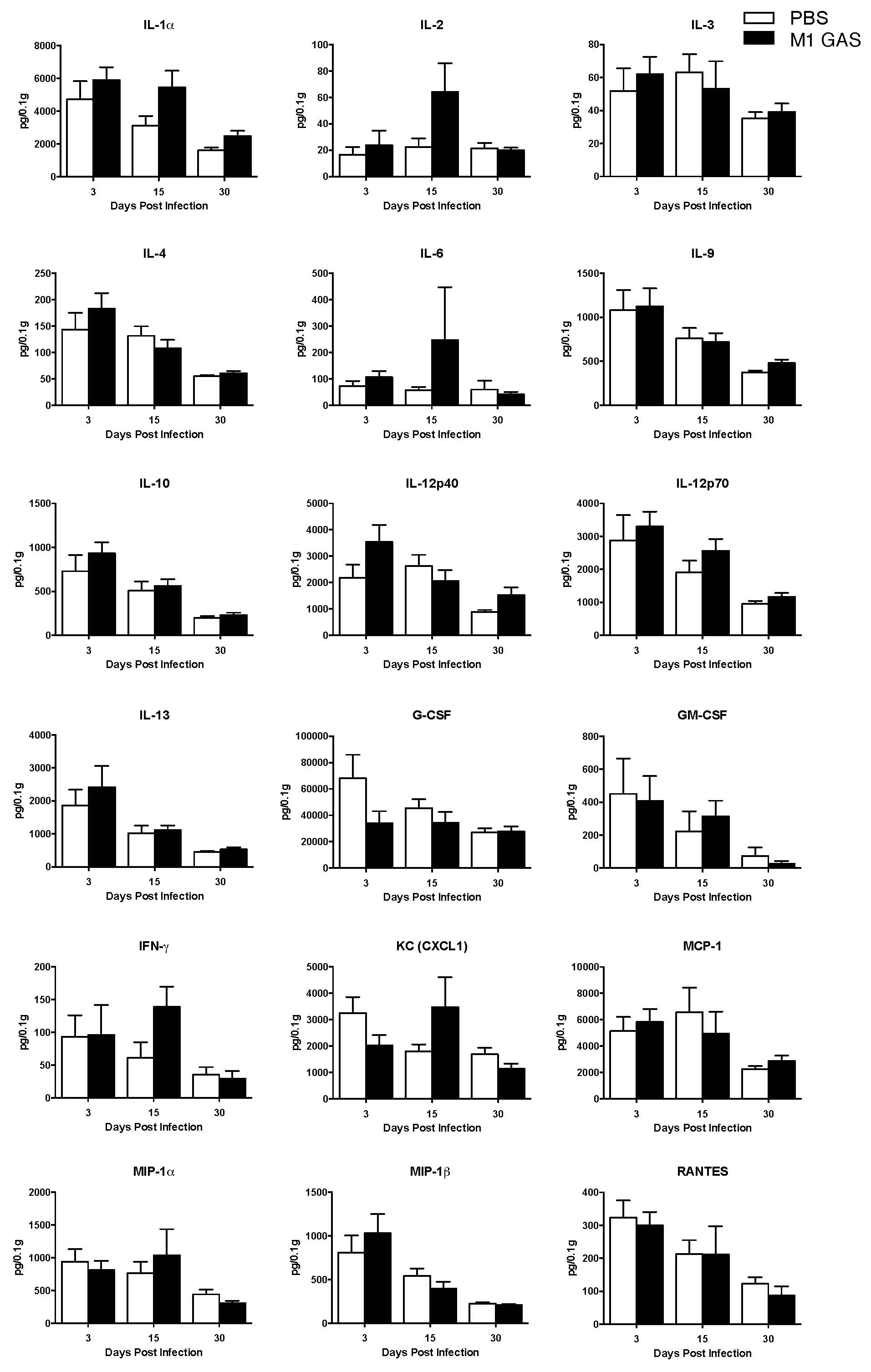
**

**Supplemental Figure 2:** Insignificant soluble inflammatory mediators in vaginal tissues of GAS infected animals. Data are mean ± standard error of the mean of 8 mice/group. Significance was measured using Mann-Whitney U-test using a p value of <0.05.


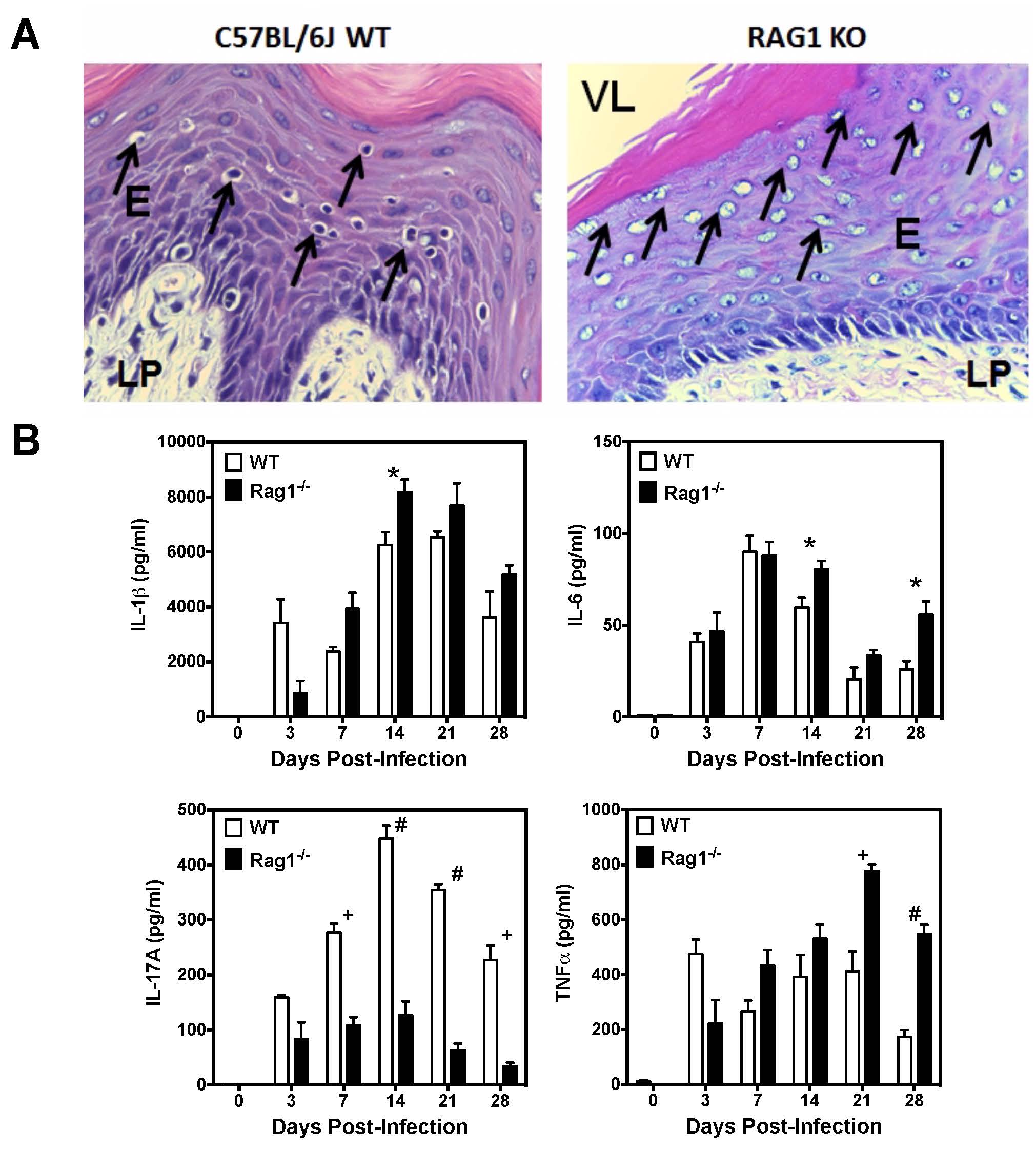


**Supplemental Figure 3:** Rag1-/- mice exhibit a normal to exaggerated innate immune response following M28 GAS vaginal colonization. Panel A shows H&E stained sections of vaginal tissue in C57BL/6J WT and Rag1-/- mice at day 14 post-infection with M28 GAS. VL = vaginal lumen, E = epithelium, LP = lamina propria, arrows indicate inflammatory cell presence, ⋅40 magnification. Panel B shows cytokine levels by ELISA of vaginal washes from WT and Rag1-/- mice post-infection with M28 GAS. Data are mean ± SEM, with at least 3 mice/point. Significance was analyzed using paired t-test using a p value of <0.05. #: p<0.001; +: p<0.01; *:p<0.05.
